# Unnecessary Frills: Communality as a Nice (But Expendable) Trait in Leaders

**DOI:** 10.3389/fpsyg.2018.01866

**Published:** 2018-10-15

**Authors:** Andrea C. Vial, Jaime L. Napier

**Affiliations:** ^1^Department of Psychology, Yale University, New Haven, CT, United States; ^2^Department of Psychology, New York University Abu Dhabi, Abu Dhabi, United Arab Emirates

**Keywords:** gender roles, gender stereotypes, leader-role expectations, agency, communality

## Abstract

Although leader role expectations appear to have become relatively more compatible with stereotypically feminine attributes like empathy, women continue to be highly underrepresented in leadership roles. We posit that one reason for this disparity is that, whereas stereotypically feminine traits are appreciated as nice “add-ons” for leaders, it is stereotypically masculine attributes that are valued as the defining qualities of the leader role, especially by men (who are often the gatekeepers to these roles). We assessed men’s and women’s idea of a great leader with a focus on gendered attributes in two studies using different methodologies. In Study 1, we employed a novel paradigm in which participants were asked to design their “ideal leader” to examine the potential trade-off between leadership characteristics that were more stereotypically masculine (i.e., agency) and feminine (i.e., communality). Results showed that communality was valued in leaders only after meeting the more stereotypically masculine requirements of the role (i.e., competence and assertiveness), and that men in particular preferred leaders who were more competent (vs. communal), whereas women desired leaders who kept negative stereotypically masculine traits in check (e.g., arrogance). In Study 2, we conducted an experiment to examine men’s and women’s beliefs about the traits that would be important to help them personally succeed in a randomly assigned leader (vs. assistant) role, allowing us to draw a causal link between roles and trait importance. We found that both men and women viewed agentic traits as more important than communal traits to be a successful leader. Together, both studies make a valuable contribution to the social psychological literature on gender stereotyping and bias against female leaders and may illuminate the continued scarcity of women at the very top of organizations, broadly construed.

## Introduction

It has been argued that stereotypically feminine traits like communality will define 21st century leaders, and women and men with these attributes will rule the future (Gerzema and D’Antonio, 2013). However, despite the embracing of so-called feminine management, women continue to be highly underrepresented in top executive roles (Catalyst, 2018), and bias against female leaders persists (Eagly and Heilman, 2016; Gupta et al., 2018). We posit that one reason for this disparity is that, whereas communality is appreciated as a nice “add-on” for leaders, it is stereotypically masculine attributes related to agency, such as competence and assertiveness, that are valued as the defining qualities of the leader role, especially by men (who are often the gatekeepers to these roles). We examined this premise in two studies in which we assessed men’s and women’s idea of a great leader with a focus on gendered attributes.

Although leadership is associated with masculine stereotypes (Schein, 1973; Koenig et al., 2011), this association appears to have weakened somewhat over time (Duehr and Bono, 2006). For example, a meta-analysis that examined the extent to which stereotypes of leaders aligned with stereotypes of men revealed that the masculine construal of leadership decreased significantly between the early 1970s and the late 2000s, as people increasingly associate leadership with more feminine relational qualities (Koenig et al., 2011). One reason for this change is the slow but noticeable surge during this period in the number of management roles occupied by women. It is possible that the raising presence of women in management roles may have reduced the tendency to associate leadership with men, given that women tend to lead differently than men (Eagly et al., 2003), and exposure to counterstereotypic individuals tends to reduce implicit biases (Dasgupta and Asgari, 2004; Beaman et al., 2009). Another reason why leadership perceptions may over time have become more androgynous (i.e., involving more stereotypically feminine in addition to stereotypically masculine qualities) is that the organizational hierarchy has flattened over time (Bass, 1999) and has come to require less directive, top-down approaches to leadership (Eagly, 2007; Gerzema and D’Antonio, 2013).

Effective leadership, which can be highly contextual (Bass, 1999), is thought to be generally participative and transformational (Bass and Riggio, 2006). Transformational leadership styles, which involve motivating, stimulating, and inspiring followers (Burns, 1978; Mhatre and Riggio, 2014), are associated with increased morale and performance at various organizational levels (Wang et al., 2011). They are also associated with female leaders somewhat more so than with male leaders (Eagly et al., 2003; Dezso and Ross, 2011; Vinkenburg et al., 2011) and tend to be viewed as relatively more feminine than autocratic or transactional styles (Stempel et al., 2015). Indeed, there is evidence that transformational leaders tend to blend masculinity and femininity and are overall more androgynous (Kark et al., 2012). Management scholars thus recognize that effective leadership combines both agency-related and communal behaviors and traits (Bass, 1999; Judge and Piccolo, 2004), which are consistently associated with men and women, respectively (Burgess and Borgida, 1999; Prentice and Carranza, 2002). Given a trend toward ever more collaborative work environments in the digital age (Bersin et al., 2017), traits and behaviors typically associated with women such as cooperation and sensitivity to others’ needs (Prentice and Carranza, 2002) are sometimes praised as the future of leadership (Gerzema and D’Antonio, 2013).

However, even though leader role expectations may be relatively more feminine today than 40 or 50 years ago (Koenig et al., 2011), women who aspire to top leadership positions continue to be at a considerable disadvantage. For example, women tend to be overrepresented in support and administrative roles (Blau et al., 2013; Hegewisch and Hartmann, 2014), but continue to occupy less than half of management positions—they comprised about 34.1% of general and operations managers in 2017 according to the Current Population Survey (U.S. Department of Labor Bureau of Labor Statistics, 2018). The proportion of women is lower in executive positions that confer major decision-making power: They occupied only 28% of chief executive roles in 2017 (U.S. Department of Labor Bureau of Labor Statistics, 2018), and a mere 5% when considering S&P 500 companies, the largest, most profitable firms in the United States (Catalyst, 2018). Although these patterns are likely to result from a variety of factors, including gender differences in interests, goals, and aspirations (Reskin et al., 1999; Diekman and Eagly, 2008; Schneider et al., 2016), there is substantial evidence that at least some of this disparity is due to gender bias (Rudman, 1998; Heilman et al., 2004; Heilman and Okimoto, 2007; Phelan et al., 2008; Rudman et al., 2012).

Bias against female leaders is likely multiply determined. On one hand, it may reflect social conservatism and antifeminist attitudes (Forsyth et al., 1997; Rudman and Kilianski, 2000; Hoyt and Simon, 2016) and a tendency to maintain the traditional status quo where women serve primarily as caretakers (Rudman et al., 2012). For example, different attitudes toward the role of women in society predict liberals’ and conservatives’ disparate levels of support for female job candidates (Hoyt, 2012). On the other hand, bias against female leaders has also been connected to the perceived relative incongruity (Eagly and Karau, 2002) or lack of fit (Heilman, 1983, 2001, 2012) between the traits typically associated with women and the traditional female gender role and the traits ascribed to the leader role. This low perceived correspondence between feminine stereotypes and leader roles makes women appear unsuitable for authority positions. Moreover, when women demonstrate the kinds of attributes that are deemed requisite for effective leadership (e.g., agency) they sometimes elicit penalties for violating gender role expectations (Heilman and Okimoto, 2007; Rudman et al., 2012; Williams and Tiedens, 2016). The effect of gender stereotypes can make it difficult for women to thrive in leadership roles (Vial et al., 2016), and can compound over time and slow women’s advancement in organizational hierarchies (Agars, 2004).

The persistence of bias against female leaders (Eagly and Heilman, 2016) appears in direct conflict with the increased valorization of more androgynous leadership styles that draw from communal, traditionally feminine traits and behaviors (Eagly, 2007; Judge and Piccolo, 2004; Judge et al., 2004; Gerzema and D’Antonio, 2013). This apparent contradiction is the focus of the current investigation, in which we test the idea that communal traits are appreciated in leaders primarily as an accessory or complement to other, more agentic qualities that tend to be viewed as more essential and defining of the leader role. We examined the trade-off that people make when thinking about agency and communality in relation to the leader role, testing the prediction that communal traits are valued in leaders only after reaching sufficient levels of agentic (i.e., more stereotypically masculine) traits. As such, even when leader role expectations may also comprise communal traits (Koenig et al., 2011), agentic traits might still be considered the hallmark of leadership—necessary and sufficient to lead. Communal attributes, in contrast, may be appreciated as nice but relatively more superfluous complements for leaders.

Moreover, even when more communal leadership styles may be increasingly appreciated (Eagly, 2007; Judge and Piccolo, 2004; Judge et al., 2004; Gerzema and D’Antonio, 2013), we propose that the people who most value it happen to be women, who are typically not the gatekeepers to top organizational positions of prestige and authority. There is meta-analytic evidence that the masculine leadership construal tends to be stronger for male versus female participants (Boyce and Herd, 2003; Koenig et al., 2011). Furthermore, compared to women, men evaluate female leaders as less ambitious, competent, intelligent, etc. (Deal and Stevenson, 1998; Vial et al., 2018), and are less likely to select female job candidates (Gorman, 2005; Bosak and Sczesny, 2011; Koch et al., 2015). Thus, the concentration of men in top decision-making roles such as corporate boards and chief executive offices (Catalyst, 2018) may be self-sustaining because men in particular tend to devalue more communal styles of leadership (Eagly et al., 1992; Ayman et al., 2009). In contrast, given that communal traits are more strongly associated with their gender in-group (Burgess and Borgida, 1999; Prentice and Carranza, 2002), women may show more of an appreciation for these traits compared to men (e.g., Dovidio and Gaertner, 1993). In the current studies, we compared men’s and women’s preferences for communality and agency in leaders.

As stated earlier, the underrepresentation of women in top leadership roles is likely to stem not only from bias against female leaders (Heilman and Okimoto, 2007) but also from women’s relatively low interest in pursuing these roles in comparison to men (Diekman and Eagly, 2008; Lawless and Fox, 2010; Schneider et al., 2016). Stereotypes linking leadership with men and communal roles with women might have a negative impact on women’s sense of belongingness and self-efficacy in leadership roles (Hoyt and Blascovich, 2010; Hoyt and Simon, 2011). For example, women report lower desire to pursue leadership roles after being exposed to stereotypic media images (Simon and Hoyt, 2013). If communal traits are overall seen as “unnecessary frills” in leaders, as we propose, and if women place higher importance on being communal when they occupy a leadership role relative to men, such mismatch might discourage women from pursuing top leadership positions (Heilman, 2001). Thus, in addition to investigating whether men and women value agency and communality differently in leaders, we also considered how much they would personally value such traits if they were to occupy a leadership role.

## Overview of Research

We conducted two studies to assess men’s and women’s idea of a great leader with a focus on gendered attributes. In Study 1, we examined the attributes that men and women viewed as requisite (vs. superfluous) for ideal leaders. In Study 2, we conducted an experiment to examine men’s and women’s beliefs about the traits that would be important to help them personally succeed in a randomly assigned leader (vs. assistant) role. In both studies, we measured trait dimensions related to gender roles and leadership including competence and assertiveness (i.e., agency) as well as communality. Agency and communality represent two basic dimensions of person perception and judgments of the self, others, and groups (Fiske et al., 2007; Abele et al., 2016). Agency is typically perceived as more self-profitable than communality, which is more often viewed as benefitting others and, as a result, communality tends to be more valued in others versus the self, whereas the reverse is true for agency (Abele and Wojciszke, 2007). Thus, it is possible that people value communality relatively more when evaluating others (vs. the self) in leadership roles. Here, we investigated how much men and women valued agency and communality when thinking about *another* in a leader role (Study 1) and when thinking of *the self* as a leader (Study 2).

Study 1 examined the notion that communal attributes are viewed as highly desirable in leaders—but only after more basic requirements have been met, which map strongly onto stereotypical masculinity (i.e., agency). Past research has examined the extent to which various attributes were seen as relevant to the leader role—either generally characteristic of leaders or typical of *successful* leaders (e.g., Schein, 1973; Powell and Butterfield, 1979; Brenner et al., 1989; Boyce and Herd, 2003; Sczesny, 2003; Sczesny et al., 2004; Fischbach et al., 2015). However, in those studies, participants rated traits one at a time and in absolute terms (e.g., “*please rate each word or phrase in terms of how characteristic it is*,” on a 5-point scale; Brenner et al., 1989, p. 664). These absolute ratings may mask the potential trade-offs between different traits when evaluating a specific person, whose traits come in bundles (Li et al., 2002).

Specifically, the importance of communal characteristics for leaders may depend on levels of other traits (Li et al., 2002, 2011; Li and Kenrick, 2006), and participants considering such traits in isolation might assume acceptable levels on other desirable attributes (e.g., agency). For example, although communality might make someone desirable as a leader, communality might be considered irrelevant if a leader is insufficiently agentic. We investigated these potential trade-offs in Study 1.

In addition to agency and communality, we included traits that were negative in valence and stereotypically masculine (e.g., arrogant) and feminine (e.g., emotional) in content. Past investigations suggest that negative masculine stereotypes, which map onto a “dominance” dimension and are related to status attainment (Cheng et al., 2013), are strongly proscribed for women (Prentice and Carranza, 2002; Hess et al., 2005). Moreover, a number of investigations have revealed that dominance perceptions play a crucial role in bias against female leaders, who are often viewed as domineering and controlling (Rudman and Glick, 1999; see also Williams and Tiedens, 2016). Similarly, a recent review suggests that negative feminine stereotypes about the presumed greater emotionality of women relative to men (Shields, 2013) are closely linked to bias against female leaders (Brescoll, 2016). For example, men in general tend to be described as more similar to successful managers in emotion expression than are women in general (Fischbach et al., 2015). Thus, in Study 1, we examined participants’ interest in minimizing these negative traits when designing their ideal leader.

In Study 2, we examined whether people’s leader role expectations differ when they think of themselves occupying that position. Many past investigations have compared perceptions of men and women in general with perceptions of *successful managers* (Schein, 1973; Powell and Butterfield, 1979; Heilman et al., 1995; Schein et al., 1996; Powell et al., 2002; Boyce and Herd, 2003; Duehr and Bono, 2006; Fischbach et al., 2015). Other studies have documented perceptions of *successful male and female managers* (Dodge et al., 1995; Heilman et al., 1995; Deal and Stevenson, 1998). We extend this prior work by directly assigning men and women to a leader role (versus an assistant role) and testing which kinds of attributes they view as important for them to be personally successful in that role. The random assignment of men and women to a leader role allowed us to draw a causal link between occupying a leadership role and differentially valuing communality and agency.

In both studies, we compared the responses of men and women, seeking to better understand how their leader-role expectations differ (Koenig et al., 2011). Past work suggests that individuals may generally prefer the kinds of attributes that are viewed as characteristic of their gender in-groups (Dovidio and Gaertner, 1993), and women compared to men have been found to possess less masculine leader-role expectations (Boyce and Herd, 2003; Koenig et al., 2011) and to value female leaders more (Kwon and Milgrom, 2010; Vial et al., 2018). Thus, we were interested in testing whether women might show higher appreciation for communal attributes in leaders in comparison to men.

## Study 1: Requisite and Superfluous Traits for Ideal Leaders

We tested the notion that communal traits are viewed as desirable in leaders—but only after more basic requirements have been met, namely, agency. We examined participants’ preferences for the kinds of traits that would characterize the ideal leader by using a methodology that was originally developed to study mate preferences (Li et al., 2002). This method essentially compares the extent to which different traits are desirable as choices become increasingly constrained, helping distinguish the attributes that are considered truly essential or fundamental in a mate (or in our case, a leader), from traits that are considered luxuries. “Luxury” traits might ultimately be superfluous if the essential attributes (or “necessities”) are not met. Conceptually, traits that are viewed as necessities tend to be favored when choices are constrained. As constraints are lifted, fewer resources are devoted to traits that are considered necessities, and more resources are allocated to luxuries.

This approach is apt to reveal the perceived trade-offs between more stereotypically feminine (i.e., communal) and masculine (i.e., agentic) leadership characteristics. By directly examining these trade-offs and identifying necessities and luxuries, we hope to clarify the seeming conflict between the increased valorization of more androgynous leadership styles that draw from traits and behaviors traditionally associated with women (Judge and Piccolo, 2004; Gerzema and D’Antonio, 2013) and the persistence of male bias (Eagly and Heilman, 2016).

We predicted that compared to communal traits, agentic traits would be rated as more of a necessity for an ideal leader, or, in other words, that communality would be treated as more of a luxury than agency. We measured two facets of agency separately, namely competence and assertiveness (Abele et al., 2016). Following Li et al. (2002), we assigned participants increasingly smaller budgets that they were instructed to use to “purchase” different traits to design their ideal leader. Participants made tradeoffs first between traits denoting competence and communality, and then between traits denoting assertiveness and communality. We expected that as people’s budgets got smaller, they would prioritize competence and assertiveness over communality.

Finally, to examine the kinds of attributes that people may find intolerable in leaders, we also included negative traits, which map onto relaxed proscriptions (Prentice and Carranza, 2002) for men (e.g., arrogant, stubborn) and women (e.g., emotional, weak). We anticipated that participants might be especially interested in minimizing negative traits that people more commonly associate with men than with women (such as arrogant) as these traits align with the culturally prevalent idea that “power corrupts” (Kipnis, 1972; Keltner et al., 2003; Inesi et al., 2012). In contrast, negative feminine stereotypes, while generally undesirable (Prentice and Carranza, 2002), are not seen as typical of those in top positions, and thus people may be less concerned with curbing these attributes when thinking about an ideal leader. Therefore, we expected to find that participants’ responses would reflect a priority to minimize negative traits more stereotypically associated with men over negative traits stereotypically associated with women.

We also considered whether participants would show more of an appreciation for positive traits that are stereotypically seen as characteristic of their gender in-group than positive stereotypes of a gender out-group (e.g., Dovidio and Gaertner, 1993). Thus, we expected female participants to rate communal traits as more necessary than male participants, whereas male participants were expected to see agentic traits (competence and assertiveness) as more necessary than female participants. These predictions also align with past research suggesting that women endorse less masculine leader stereotypes than men (Boyce and Herd, 2003; Koenig et al., 2011) and are more supportive of female leaders (Kwon and Milgrom, 2010; Vial et al., 2018). Additionally, participants were expected to show less of an aversion for negative traits that are stereotypical of their gender in-group than negative stereotypes of a gender out-group—that is, we expected female participants to see it as more of a priority to reduce negative traits commonly associated with men than male participants, whereas male participants were expected to prioritize minimizing negative feminine stereotypes more so than female participants.

### Method

#### Participants

Power analysis performed with G^∗^Power 3.1 (Faul et al., 2007) indicated the need for at least 162 participants to have adequate power (1-β = 0.80) to detect small to medium effect sizes (*f* = 0.175) for the main effects of budget, participant gender, and their interaction for each of three lists of traits. In total, 281 participants took part in the study via Amazon Mechanical Turk (Mturk). The study was described to potential Mturk participants (i.e., those with at least 85% approval rates) as a short survey on work-related attitudes and impressions of other people, in which participants would be asked to read some materials and answer some questions about their experiences, beliefs, and attitudes. The study took approximately 5 minutes and participants were compensated $0.55. Eight participants (2.8%) indicated that some of their answers were meant as jokes or were random. We report analyses excluding these 8 participants (*n* = 273; mean age = 35.94, *SD* = 11.73; 57.5% female; 76.2% White). One participant did not indicate gender (0.4%).

#### Procedure

Participants were asked to think about the attributes that would make someone an ideal leader. We asked them to design their ideal leader by purchasing traits from three different lists, and we gave participants a set budget of “leader dollars” that they could spend at their discretion. Each of the three lists contained 10 traits in random order, and participants could spend up to 10 dollars on each trait. For each list of traits, participants were first asked to allocate 60 leader dollars between the 10 traits. Then, participants were asked to do this exercise again two more times, first with a budget of 40 leader dollars, and then with a budget of 20 leader dollars. All stimuli are reported in full in **Appendix A**.

The first list of traits included five agentic/competence traits (capable, competent, confident, common sense, intelligent) and five communal traits (good-natured, sincere, tolerant, happy, trustworthy). The second list included five agentic/assertive traits (ambitious, assertive, competitive, decisive, self-reliant) and an additional five communal traits (cooperative, patient, polite, sensitive, cheerful). The third list included five negative masculine stereotypes (arrogant, controlling, rebellious, cynical, stubborn) and five negative feminine stereotypes (emotional, naïve, shy, weak, yielding), as classified by Prentice and Carranza (2002). The instructions for the third list were slightly different from the first two lists, as participants were asked to indicate how much they would pay so that their ideal leader would *not possess* each of the 10 negative traits. At the end of the study, all participants were asked basic demographic questions (e.g., age, race), and received a debriefing letter. In both studies, prior to debriefing, we asked participants to indicate whether any of their answers were random or meant as jokes (“yes” or “no”). We reassured participants that they would receive full compensation regardless of their answers to encourage honest responding.

### Analytic Strategy

We first computed the proportion of each overall budget that was allocated to agency/competence versus communality, agency/assertiveness versus communality, and negative masculine versus feminine stereotypes. For the first two, we combined the amounts allocated to agentic traits (competence or assertiveness) for each budget and computed the total proportion such that higher scores indicated a larger proportion of the budget was allocated to agency (competence or assertiveness) versus communality. We followed the same procedure for the negative traits, where higher scores indicated a larger proportion of the budget allocated to eliminate negative traits stereotypically associated with men over those associated with women.

As the budget expands, people allocate an increasingly smaller proportion of their extra income to necessities and spend a larger proportion of income on luxuries. In order to investigate which trait categories were seen as necessities and which were seen as luxuries, we followed Li et al.’s (2002) analytic strategy and compared participant allocations in the low budget (i.e., 20 leader dollars) with how they allocated their last 20 leader dollars. We computed the allocation of the last 20 dollars by subtracting the amount purchased in the medium budget (40 dollars) from that of the high budget (60 dollars), and then divided by 20. This strategy is similar to asking participants how they would allocate an additional 20 leader dollars after they have already spent 40. We submitted the proportion scores for the first 20 and the last 20 leader dollars as repeated measures in three separate Analysis of Variance (ANOVA) tests, one for each trait category (i.e., competence/communality, assertiveness/communality, and negative masculine/feminine stereotypes), with participant gender as between-subjects factor.

### Results

We examined the bivariate associations between the proportion of budgets allocated to the different sets of traits at the three budget levels. Across budgets, the proportion spent to gain competence (vs. communality) was significantly positively associated with the proportion spent to gain assertiveness (vs. communality) (correlations ranging from *r* = 0.47 to *r* = 0.39, all *p*s < 0.001, depending on budget.) Additionally, across budgets, the proportion spent to gain competence (vs. communality) was significantly negatively associated with the proportion spent to minimize negative traits that are more stereotypically masculine (vs. feminine) (correlations ranging from *r* = -0.33 to *r* = -0.15, all *p*s < 0.001, depending on budget). The same pattern emerged even more strongly for the association between the proportions spent to gain assertiveness (vs. communality) and the proportions spent to minimize negative traits that are more stereotypically masculine (vs. feminine) (correlations ranging from *r* = -0.47 to *r* = -0.41, all *p*s < 0.001, depending on budget). In other words, these bivariate correlations suggest that a stronger preference for agency (competence or assertiveness) over communality was associated with a weaker desire to reduce negative masculine traits over negative feminine traits. (Partial correlations controlling for participant gender revealed the same patterns).

#### Competence Versus Communality

There was a significant effect of budget for the competence/communality traits list, *F*(1,270) = 2780.21, *p* < 0.001, η_p_^2^ = 0.911, such that the difference in the proportion allocated to competence relative to communality was higher for the first 20 dollars (*M* = 0.59, *SD* = 0.18) compared to the last 20 dollars (*M* = -0.001, *SD* = 0.004), *M_D_* = 0.60, *SE* = 0.011, 95% CI[0.573, 0.618]. This pattern is consistent with participants viewing competence as more of a necessity and communality as more of a luxury. There was also a significant main effect of participant gender, *F*(1,270) = 5.50, *p* = 0.020, η_p_^2^ = 0.020, and a significant interaction between participant gender and budget, *F*(1,270) = 5.51, *p* = 0.020, η_p_^2^ = 0.020. Men and women differed in their allocation of their first 20 dollars, such that men prioritized competence over communality (*M* = 0.62, *SD* = 0.19), to a significantly higher extent than women (*M* = 0.57, *SD* = 0.17), *M_D_* = 0.05, *SE* = 0.022, 95% CI[0.008, 0.097], *p* = 0.020, η_p_^2^ = 0.020. However, men and women allocated the last 20 dollars in a similar way, *M_D_* = -0.001, *SE* = 0.001, 95% CI[0.000, 0.002], *p* = 0.290, η_p_^2^ = 0.004.

**FIGURE 1:**
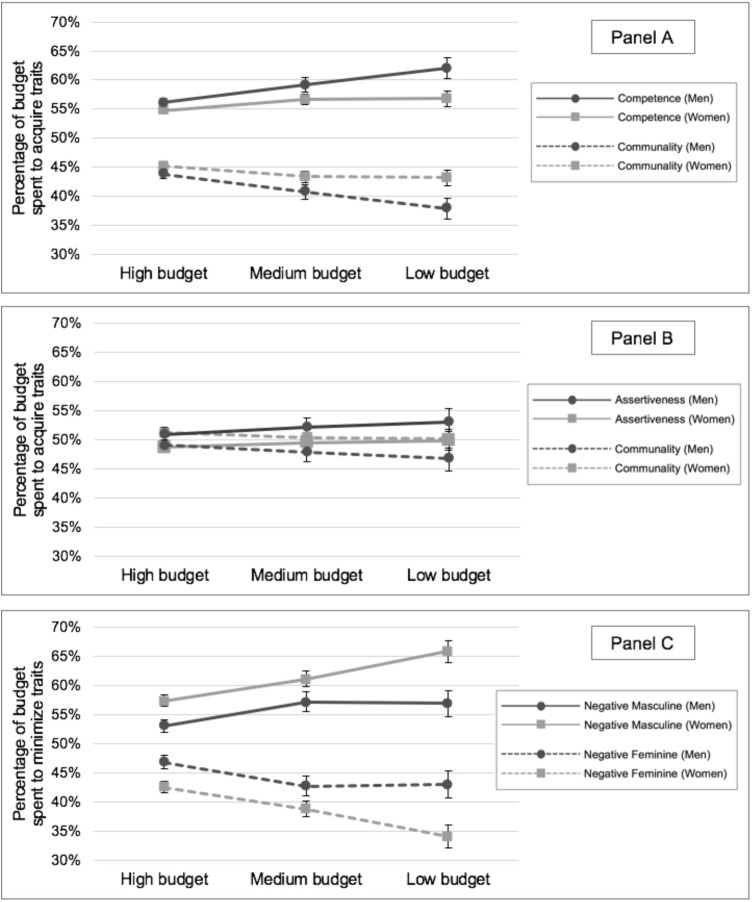
Ideal percentages for the three trait categories as a function of budget and participant gender in Study 1. Error bars represent the standard error of the mean. **(A)** Ideal percentages of the budget allocated to maximizing competence versus communality. **(B)** Ideal percentages of the budget allocated to maximizing assertiveness versus communality. **(C)** Ideal percentages of the budget allocated to minimizing negative masculine versus feminine stereotypes.

The ideal proportions of competence/communality as a function of budget are presented in **Figure 1A**. As can be seen in the figure, for all three budgets, male as well as female participants spent more on competence traits than on communal traits, and this difference became larger as options became more constrained (i.e., as the budget became smaller). While men’s and women’s allocations were more similar for the high and medium budgets, when the budget became smaller, men’s preference for competence over communality (62% vs. 38% of the budget) was stronger than women’s (57% vs. 43% of the budget). In other words, the tendency to view competence as more of a necessity than communality was apparent in both men and women, and men valued competence over communality more strongly than women when choices were constrained.

#### Assertiveness Versus Communality

There was also a significant effect of budget for the assertiveness/communality traits list, *F*(1,270) = 1428.82, *p* < 0.001, η_p_^2^ = 0.841, such that the difference in the proportion allocated to assertive over communal traits was significantly higher for the first 20 dollars (*M* = 0.51, *SD* = 0.22) compared to the last 20 dollars (*M* = -0.0005, *SD* = 0.005), *M_D_* = 0.51, *SE* = 0.014, 95% CI[0.488, 0.542]. There was no significant main effect of gender, *F*(1,270) = 1.49, *p* = 0.223, η_p_^2^ = 0.005, and, contrary to predictions, the interaction between budget and participant gender was not significant, *F*(1,270) = 1.45, *p* = 0.229, η_p_^2^ = 0.005. The ideal proportions of assertive versus communal traits as a function of budget are presented in **Figure 1B**. As can be seen in the figure, as the budget became smaller, participants spent slightly but reliably more on assertive traits than on communal traits. This pattern is consistent with participants viewing assertiveness as more of a necessity and communality as more of a luxury.

#### Negative Masculine/Feminine Stereotypes

Finally, there was a significant effect of budget for the last list of traits focused on negative masculine/feminine stereotypes, *F*(1,270) = 1760.12, *p* < 0.001, η_p_^2^ = 0.867. The difference in the proportion allocated to minimizing negative masculine stereotypes relative to negative feminine stereotypes was higher for the first 20 dollars (*M* = 0.62, *SD* = 0.24) compared to the last 20 dollars (*M* = -0.002, *SD* = 0.005), *M_D_* = 0.62, *SE* = 0.015, 95% CI[0.586, 0.646]. This pattern is consistent with participants viewing the minimization of negative masculine stereotypes as more of a necessity and the minimization of negative feminine stereotypes as more of a luxury. There was a significant main effect of participant gender, *F*(1,270) = 9.22, *p* = 0.003, η_p_^2^ = 0.033, and a significant interaction between participant gender and budget, *F*(1,270) = 8.74, *p* = 0.003, η_p_^2^ = 0.031. Men and women differed in their allocation of the first 20 dollars, such that women prioritized the minimization of negative masculine over feminine stereotypes (*M* = 0.66, *SD* = 0.24) to a significantly higher extent than men (*M* = 0.57, *SD* = 0.24), *M_D_* = 0.09, *SE* = 0.030, 95% CI[0.031, 0.148], *p* = 0.003, η_p_^2^ = 0.032. Men and women allocated the last 20 dollars in a similar way, *M_D_* = 0.001, *SE* = 0.001, 95% CI[-0.001, 0.001], *p* = 0.770, η_p_^2^ < 0.001.

The ideal proportions of negative masculine/negative feminine stereotypes as a function of budget are presented in **Figure 1C**. As can be seen in the figure, for all three budgets, male as well as female participants spent higher proportions of their budgets to minimize negative masculine stereotypes than to minimize negative feminine stereotypes, and this difference became larger as options became more constrained (i.e., as the budget became smaller). While women and men’s allocations were more similar for the high and medium budgets, when the budget became smaller women’s interest in minimizing negative masculine stereotypes relative to negative feminine stereotypes (66% vs. 34% of the budget) was stronger than men’s (57% vs. 43% of the budget). In other words, the tendency to see it as a necessity to curb negative masculine (vs. feminine) stereotypes was apparent in both men and women, and women devalued negative masculine (vs. feminine) stereotypes more strongly than men when choices were constrained.

### Discussion

The goal of Study 1 was to examine the attributes that men and women view as requisite (vs. superfluous) for ideal leaders. As predicted, leader agency was seen as more of a necessity relative to leader communality, which was viewed as more of a luxury. We found that when people’s budgets were constrained, both men and women were more likely to give up communality in favor of both competence and assertiveness.

It is worth noting that, when participant choices were only minimally constrained (i.e., in the high budget condition), the relative preference for assertiveness over communality appeared to reverse. In other words, when they could choose rather freely, participants in this study favored a communal leader over an assertive one. Such reversal is in line with the increased valorization of more androgynous leadership styles that draw from traditionally feminine traits and behaviors (Judge and Piccolo, 2004; Judge et al., 2004; Eagly, 2007; Gerzema and D’Antonio, 2013). However, the methodology employed clearly indicates that communal traits do not hold the same value as assertiveness in relation to idealized leadership, as communal traits were only valuable once agentic attributes had been sufficiently met.

We found that participants devoted a larger proportion of their budgets to minimizing negative masculine stereotypes, such as arrogant and controlling, than negative feminine stereotypes, such as emotional. This preoccupation with negative masculine stereotypes in particular may reflect a general view that power corrupts (Kipnis, 1972; Keltner et al., 2003; Inesi et al., 2012), as well as an attempt to keep those deleterious effects of power at bay in ideal leaders. In contrast, minimizing negative feminine stereotypes became of interest only after negative masculine stereotypes were sufficiently reduced.

Although both men and women ultimately preferred agency to communality, the results suggest that, compared to men, women prefer leaders who show more of a balance between competence and communality (whereas men more strongly favor competence), and who can keep traits like arrogance or stubbornness in check. In line with our expectation that participants would be more tolerant of negative stereotypes of their gender in-group than negative stereotypes of a gender out-group, we found that women in particular prioritized minimizing masculine negative stereotypes when thinking about an ideal leader. Men seemed more tolerant of these negative traits, which are generally seen as more typical in their gender in-group than the gender out-group (Prentice and Carranza, 2002). Instead, men spent relatively more of their budgets to curb negative feminine stereotypes in leaders.

A potential limitation in Study 1 is that, in the absence of a qualifier, participants might have thought primarily about a male individual when designing their ideal leader—given that these roles historically have been (and continue to be) disproportionally occupied by men (Blau et al., 2013), and given a general tendency to think of men as category exemplars, as reviewed recently (Bailey et al., 2018). Rather than asking participants to design their ideal “female” or “male” leader, which may arouse socially desirable responses, we again examined which traits people think are necessary for leadership in Study 2 by having male and female participants imagine themselves in a leadership (or assistant) role, and then asking them to rate what traits they believe are important to succeed in that role.

## Study 2: Important Traits to Succeed in Leader Vs. Assistant Roles

In Study 2, we had participants imagine themselves in either a leadership or assistantship role and examined the extent to which they believed they would need to act in agentic and communal ways in order to be successful in that role. To our knowledge, the present study was the first one to examine adult men’s and women’s beliefs about the traits they would need to be successful in a randomly assigned leader role. As such, this study is particularly well suited to establish a direct causal link between occupying a leadership role and differentially valuing agentic and communal traits.

We expected that agentic traits, including competence and assertiveness, would be rated as more important to succeed in a leader role, but as less crucial for assistant roles. In contrast, we expected participants to see communal traits, such as patient and polite, as more important to be a successful assistant than a successful leader. Moreover, although previous research has shown that agency is more desirable than communality in the self (as compared to in others) (Abele and Wojciszke, 2007), we predict that the role will influence the extent to which people find agentic traits desirable in the self. Specifically, whereas we expected that agency would take precedence over communality for participants in the leader role, we expected to find the reverse for those in the assistant role, for whom communality would take precedence over agency.

We anticipated that both male and female participants would rate agentic traits (like competence and assertiveness) as more important to succeed as a leader than communality, similar to past investigations (Koenig et al., 2011). However, we also anticipated an interaction between role and participant gender, such that women compared to men would rate communal traits as more important to succeed as a leader. This is because people tend to favor traits and attributes that are characteristic of their in-groups (versus attributes that are not, or that characterize an outgroup) (Dovidio and Gaertner, 1993), and because women compared to men have been found to possess less masculine leader-role expectations (Boyce and Herd, 2003; Koenig et al., 2011) and to value female leaders more (Kwon and Milgrom, 2010; Vial et al., 2018).

### Method

#### Participants

The study employed a 2×2×3 mixed design with participant gender (male vs. female) and role condition (leader vs. assistant) as between-subjects factors and trait category (competence, assertiveness, and communality) as a within-subjects factor. We enrolled 252 MTurk participants with a HIT completion rate of 95% or higher, who were compensated $0.55. The study took approximately 10 minutes and was described to potential participants as a research study about personal experiences, feelings, and attitudes. Three participants (1.2%) indicated that some of their answers were meant as jokes or were random. We report analyses excluding these 3 participants (*n* = 249; mean age = 32.55, *SD* = 11.88; 42.6% female; 71.9% White). One participant (0.4%) did not indicate gender. A sensitivity power analysis using G^∗^Power 3.1 (Faul et al., 2007) showed a sample of this size (*n* = 249) is sufficient to detect a small interaction effect between within- and between-factors, i.e., *f*(U) = 0.169 with power = 0.80 and *f*(U) = 0.208 with power = 0.95 (assuming α = 0.05, four groups, and 3 repeated measures).

#### Procedure

Participants first read a short vignette asking them to imagine that they were part of a team working on an important project. The full text of the vignette is presented in **Appendix B**. Half of participants were randomly assigned to a role condition in which they imagined being the team leader, and the other half were assigned to a role condition in which they imagined being the assistant to the leader. All participants were asked to indicate how important each of a series of attributes was to be successful in their role. Specifically, for each trait, they read “As [a leader/an assistant] it is important to be [trait],” and indicated their answer from 1 (*not at all*) to 7 (*extremely so*). The list of traits, all of which were used in Study 1, included eight agentic traits, three of which measured competence (i.e., competent, confident, capable; α = 0.75), and five of which measured assertiveness (i.e., ambitious, assertive, competitive, decisive, self-reliant; α = 0.78), and eight communal traits (i.e., cheerful, cooperative, patient, polite, sensitive, tolerant, good-natured, sincere; α = 0.83).^1^Finally, all participants were asked basic demographic questions (e.g., age, race), and received a debriefing letter.

### Results

We conducted a mixed-model ANOVA with participant gender and experimental role condition as between-subjects factors, and trait category (competence, assertiveness, and communality) as a repeated measure. As expected, we found a significant interaction between role and trait category, *F*(2,243) = 32.31, *p* < 0.001, η_p_^2^ = 0.210. The interaction between participant gender and trait category was not significant, *F*(2,243) = 1.85, *p* = 0.159, nor was the 3-way interaction between trait category, role, and participant gender, *F*(2,243) = 1.19, *p* = 0.306.

**FIGURE 2:**
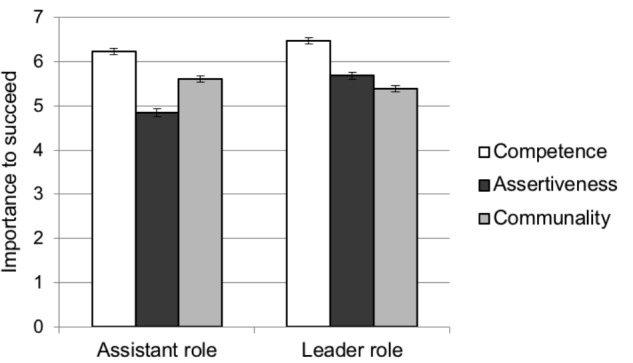
Mean ratings of importance to succeed in a randomly assigned assistant versus leader role for all trait dimensions in Study 2. Error bars represent the standard error of the mean.

All means are represented in **Figure 2**.

Pairwise comparisons revealed that participants in the leader role rated both competence, *M_D_* = 0.242, *SE* = 0.09, 95% CI [0.056, 0.428], *p* = 0.011, and assertiveness, *M_D_* = 0.839, *SE* = 0.12, 95% CI [0.599, 1.078], *p* < 0.001, as significantly more important to succeed compared to participants in the assistant role. In contrast, communality was rated as significantly more important to succeed as an assistant than as a leader, *M_D_* = -0.218, *SE* = 0.10, 95% CI [-0.422, -0.013], *p* = 0.037.

Looking at it another way, participants in both the leader and assistant roles rated competence as the most important set of traits, higher than assertiveness (*M_D_* = 0.794, *SE* = 0.09, 95% CI [0.627, 0.961], *p* < 0.001 in leader role; and *M_D_* = 1.391, *SE* = 0.09, 95% CI [1.223, 1.559], *p* < 0.001 in assistant role) and communality (*M_D_* = 1.085, *SE* = 0.07, 95% CI [0.945, 1.226], *p* < 0.001 in leader role; and *M_D_* = 0.626, *SE* = 0.07, 95% CI [0.485, 0.766], *p* < 0.001 in assistant role). Those in the leader condition rated assertiveness as more important than communality, *M_D_* = 0.291, *SE* = 0.09, 95% CI [0.108, 0.474], *p* = 0.002, whereas those in the assistant condition did the reverse, rating communal traits as more desirable than assertive ones, *M_D_* = -0.765, *SE* = 0.09, 95% CI [-0.949, -0.581], *p* < 0.001.

### Discussion

The goal of Study 2 was to examine men’s and women’s beliefs about the traits that would be important to help them personally succeed in a randomly assigned leader (vs. assistant) role. As expected, results supported our general predictions. In line with past work (Koenig et al., 2011), people rated competence and assertiveness as more necessary for success as a leader (vs. assistant), and communality as more necessary for success as an assistant (vs. leader). Although competence was seen as relatively more important for leaders than for assistants (as would be expected for a high-status professional role; e.g., Magee and Galinsky, 2008; Anderson and Kilduff, 2009), competence emerged as the most important trait to succeed in both types of roles. Moreover, as we had anticipated, even though people tend to value agency over communality when thinking of the self (Abele and Wojciszke, 2007), role assignment had the effect of reversing this pattern for participants in the assistant role (at least in terms of assertiveness, which assistants rated as less important for them to succeed than communality).

Even though we had expected to find that women (vs. men) would value communal traits to a higher extent (Boyce and Herd, 2003; Koenig et al., 2011), women were just as likely as men to see these traits as relatively unimportant for them personally to be successful in leader roles, and we failed to find any participant gender effects either in the leader or assistant role. This null interaction effect—which stands in contrast to the gender differences we observed in Study 1—might reflect the power of role demands to change self-views (Richeson and Ambady, 2001) and to override the influence of other factors such as category group memberships (LaFrance et al., 2003). Moreover, it is possible that, even if women valued communality more so than men when thinking about *other* leaders, they may nevertheless feel as though acting in a stereotypically feminine way and behaving less dominantly than a traditional male leader would place them at a disadvantage relative to men (Forsyth et al., 1997; Bongiorno et al., 2014). Such self-versus-other discrepancy might explain why the expected gender difference in the appreciation of communality relative to agency-assertiveness emerged in Study 1, when participants were thinking of ideal leaders, but was not apparent in Study 2, when participants were asked to think about themselves in a leader role.

## General Discussion

The main goal of this investigation was to examine people’s beliefs about what makes a great leader with a focus on gendered attributes, given that more stereotypically feminine leader traits (i.e., communality) appear to have become more desirable over time (Koenig et al., 2011), and that some have claimed that these attributes will define the leaders of the future (Gerzema and D’Antonio, 2013). The results of the two studies reported here were generally in line with our predictions that men’s and women’s idea of what it takes to be successful in leadership roles is essentially agency, which is a stereotypically masculine attribute. Communality is appreciated in leaders, but only as a non-vital complement to the fundamentally masculine core of the leader role. Whereas past investigations have reached similar conclusions (e.g., Koenig et al., 2011), the current studies contribute to this body of work in important ways.

This investigation was the first that we know of to examine the potential trade-off between agentic and communal traits in leaders. The results of Study 1 supported the proposed view that communality is valued in leaders only after meeting the more stereotypically masculine requirements of being competent and assertive. Importantly, the methods in Study 1 revealed that communal traits are indeed valued in leaders when choices are unconstrained. These results indicate that when participants rate traits independently from one another, as in past studies (e.g., Schein, 1973; Powell and Butterfield, 1979; Brenner et al., 1989; Boyce and Herd, 2003; Sczesny, 2003; Sczesny et al., 2004; Fischbach et al., 2015), their responses might unduly inflate their true appreciation for communal leader attributes. When choices were constrained, participants in Study 1 showed a clear preference for agentic leader traits (i.e., competence and assertiveness). Other investigations have similarly revealed how subtle differences in the measurement of group stereotypes may change the overall conclusions (Biernat and Manis, 1994). We hope that the methods in Study 1 may be adapted in future investigations to further examine gender leader-role expectations and preferences.

Moreover, the random assignment of men and women to a leader (vs. assistant) role in Study 2 allowed us to establish a direct causal link between occupying a leadership position and differentially valuing agentic and communal traits, extending past investigations (e.g., Heilman et al., 1995; Boyce and Herd, 2003; Duehr and Bono, 2006; Fischbach et al., 2015). We found that men and women were largely in agreement; both indicated that it would be more important for them to possess agentic rather than communal traits in order to be a good leader. These results underscore women’s internalization of stereotypically masculine leader role expectations, which could discourage women from pursuing leadership roles (Bosak and Sczesny, 2008; Latu et al., 2013; Hoyt and Murphy, 2016). Furthermore, if women tend to internalize a stereotypically masculine view of leadership, it follows that women who have an interest in and attain leadership roles might have a strong tendency to behave in line with those role expectations—for example, by displaying assertiveness, which could elicit backlash and penalties for violating gender prescriptions (Rudman and Glick, 1999; Phelan et al., 2008).

Alternatively, it is possible that, even though women may value communality in leaders more so than men, as Study 1 revealed, they may nevertheless feel as though enacting these characteristics would make them appear less effective as leaders or place them at a disadvantage relative to male leaders (Forsyth et al., 1997; Bongiorno et al., 2014). For example, past investigations suggest that female leaders who behave in relatively less agentic ways are perceived to be less likable and less influential than similar male leaders (Bongiorno et al., 2014). This differentiation between the traits that women value in leaders and the traits they feel as though they must exhibit to be successful in that role (perhaps to be taken seriously by others in that role; Yoder, 2001; Chen and Moons, 2015) may explain why we did not find the predicted interaction with participant gender in Study 2.

## Limitations and Remaining Questions

Although the random assignment of men and women to a leader (vs. assistant) role in Study 2 allowed us to extend past investigations by drawing causal links between roles and trait desirability, a potential limitation in our approach is that the role manipulation may also conceivably lead to a difference in psychological feelings of power across conditions (Anderson and Berdahl, 2002; Schmid Mast et al., 2009). Given the large conceptual overlap between leadership and “power” (commonly defined as asymmetric control over resources; Keltner et al., 2003), it is possible that the results of Study 2 reflect at least in part the way men and women feel when they are in a position of power, independently from their role as leaders or assistants. Future investigations may address this issue by measuring felt power (Anderson et al., 2012) to examine whether participants value similar traits as they did in Study 2 over and above felt power. For example, it is conceivable that individuals in leadership roles that foster stronger (vs. weaker) feelings of power might value communality to a lower extent, and behave more dominantly overall (e.g., Tost et al., 2013).

Another potential limitation in Study 2 is that participants assigned to the assistant role condition might have assumed that the team leader was male—consistent with the notion that people think “male” when they think “manager” (Schein, 1973). Therefore, it is unclear whether the traits that they thought would help them be a successful assistant would be contingent on the assumption that they would be assisting a male-led team. Future investigations may probe whether people believe that it takes different attributes to successfully work for a female versus a male leader, and how those beliefs impact their support for male and female supervisors. For example, if men think that a female leader would expect more cooperation from subordinates than a male leader, this expectation may partly explain their reluctance to work for women.

It is also worth noting that, in both studies, we did not specify the context under which leadership (and, in Study 2, assistantship) was taking place. It seems likely that participants were thinking of some traditionally male-dominated domain (as businesses typically are). However, one important next step for future work is to examine whether the leadership domain affects which traits people value in leaders, and which traits they would find valuable for them, personally, to be a successful leader. Leaders tend to be considered particularly effective in industries and domains in which the gender composition is congruent with the gender of the leader (Ko et al., 2015; see also Eagly et al., 1995). It is conceivable that being the leader of a team that is working in a traditionally feminine domain (e.g., childcare, nursing, or even a business that caters primarily to women, such as maternity-wear or cosmetics) might change people’s perception of which traits are most important.

Whereas our investigation was focused on the general dimensions of agency and communality (Abele et al., 2016), future research might adapt the methodology of Study 1 to examine the potential tradeoffs between other kinds of leader attributes. For instance, past research has examined task-oriented versus person-oriented trait dimensions (Sczesny et al., 2004), traits related to activity/potency (e.g., forceful, passive; Heilman et al., 1995), “structuring” versus “consideration” behaviors (Cann and Siegfried, 1990; Sczesny, 2003), and transformational leader traits (Duehr and Bono, 2006), to name a few. In particular, given that transformational leadership styles tend to be quite favorable in contemporary organizations (Wang et al., 2011), and are more closely associated with femininity (Kark et al., 2012; Stempel et al., 2015), it would be especially interesting to examine whether such transformational leader attributes are also considered “unnecessary frills” (much like communal attributes in Study 1). As mentioned earlier, the context of leadership (more male- vs. more female-dominated) may be an important moderating factor worthy of consideration (Ko et al., 2015). For example, male followers appear to react more negatively to transformational leadership styles compared to female followers (Ayman et al., 2009). Thus, it is possible that the tradeoff between more and less transformational leadership attributes may partly depend on the specific industry or domain.

Similarly, whereas we examined two sub-dimensions of agency (i.e., competence and assertiveness) following Abele et al. (2016), we did not distinguish different facets within the dimension of communality. Specifically, research suggests that communality may be broken into sub-dimensions of warmth or sociability (e.g., friendly, empathetic) and morality (e.g., fair, honest) (Abele et al., 2016), a distinction that may be meaningful and consequential in the evaluation of leaders. It has been argued that morality in particular, more so than warmth/sociability, plays a primary role in social judgment (Brambilla et al., 2011; Brambilla and Leach, 2014; Leach et al., 2017), and moral emotions are implicated in bias against agentic female leaders (Brescoll et al., 2018). Thus, future investigations may examine how the tradeoff between agency and communality explored in our research might change when the morality facet of communality is considered separately from the warmth/sociability facet.

Additional research may extend the current investigations by adapting the methodology we employed in Study 1 (which we, in turn, adapted from Li et al., 2002) in various ways to further examine leader-role expectations and preferences for communality and agency in leaders (both in others and in the self). Whereas we did this in the current investigation by testing the potential tradeoffs between ideal levels of communal and agentic traits (Study 1) and the extent to which men and women viewed those traits as personally important to succeed in a leader (vs. assistant) role (Study 2), it would be worthwhile to merge these two paradigms in the future. For example, men and women in leadership roles might be asked to think about the traits they would need to be successful and then to “purchase” various amounts of those traits for themselves. Similarly, participants could be asked to purchase traits to design the ideal leader versus the ideal subordinate (e.g., the perfect assistant).

## Implications and Conclusion

The findings from this investigation may illuminate the continued scarcity of women at the very top of organizations, broadly construed (Eagly and Heilman, 2016; Catalyst, 2018). Overall, across studies, both women and men saw communality as relatively unimportant for successful leadership. These traits, however, make women particularly well suited to occupy low status positions (Study 2), which may contribute to gender segregation (Blau et al., 2013) via women’s self-selection into low status roles (Diekman and Eagly, 2008; Schneider et al., 2016).

On a more positive note, our results also suggest that women may be more supportive than men of leaders who exhibit more feminine leadership styles. We found as we had expected that women showed higher appreciation for communal attributes in leaders in comparison to men (Study 1). Furthermore, in Study 1 we also examined participants’ interest in minimizing negative traits stereotypically associated with men and women when designing their ideal leader. Rather than desiring leaders to possess lower amounts of negative traits that are more stereotypically feminine (such as emotional; Shields, 2013), participants desired leaders to lack negative traits more commonly associated with men (like arrogance; Prentice and Carranza, 2002), and this preference was stronger among women compared to men.

Whereas many studies have assumed to some extent that descriptive gender and leader stereotypes are similarly shared by men and women (see review by Rudman and Phelan, 2008), our results suggest that this assumption needs to be reconsidered, particularly with respect to gender traits that are relevant to leadership. Even when men and women agreed on the attributes they would personally need to be successful leaders (Study 2), Study 1 showed that women ideally prefer leaders who are more communal relative to men, and that they feel more negative than men about certain aspects believed to characterize both men and leaders (arrogance). These subtle gender differences in leader-role expectations dovetail past investigations showing patterns consistent with gender in-group favoritism effects (Tajfel et al., 1971; Greenwald and Pettigrew, 2014) on evaluation of female and male authorities (Eagly et al., 1992; Norris and Wylie, 1995; Deal and Stevenson, 1998; Ayman et al., 2009; Kwon and Milgrom, 2010; Bosak and Sczesny, 2011; Paustian-Underdahl et al., 2014; Vial et al., 2018). For example, past studies have revealed that women have more positive attitudes toward female authorities compared to men, whether implicit (Richeson and Ambady, 2001) or explicit (Rudman and Kilianski, 2000). Similarly, a recent investigation revealed that female employees working for female supervisors tend to respect those supervisors more so than male employees and engage in positive work behaviors more frequently than male employees when working for a woman (Vial et al., 2018).

Overall, the two studies reported here further suggest that women might be relatively more supportive of leaders with more communal leadership styles compared to men. Thus, while it may be too soon to tell whether these stereotypically feminine traits will indeed define the leaders of the 21^st^ century (Gerzema and D’Antonio, 2013), our investigation suggests that women might be more willing than men to embrace this trend.

## Ethics Statement

This study was carried out in accordance with the recommendations of the American Psychological Association’s Ethical Principles in the Conduct of Research with Human Participants. The protocol was approved by the Institutional Review Board at Yale University. All subjects gave written informed consent in accordance with the Declaration of Helsinki.

## Author Contributions

AV wrote the first draft of the manuscript. JN provided feedback and edits. Both authors worked collaboratively on study design and data collection and analysis.

## Conflict of Interest Statement

The authors declare that the research was conducted in the absence of any commercial or financial relationships that could be construed as a potential conflict of interest.

## Footnotes

1 The following traits were added for exploratory purposes but were not included in Study 1 or in the analyses for Study 2: excitable, sophisticated, refined, immoral, self-interested, cut-off, cynical, visionary, inspiring, dominant, powerful, independent. After rating all traits, participants indicated how much they identified with their gender in-group. Gender identification did not vary with condition or participant gender and was excluded from all analyses.

## Appendix A Study 1 Stimuli

Participants first read the following preliminary instructions:

Please take a moment to think about the characteristics that would make someone your ideal leader. By “leader” we mean someone within a group who:

Controls group resources. Hires, promotes, and fires group members. Determines what needs to be done in order to achieve the group’s goals. Assigns tasks to group members. Evaluates group members’ performance.

Ultimately, a leader is responsible for the group’s outcomes. In this study, we will ask you to “design” your IDEAL LEADER by purchasing traits from a predetermined list. We will give you a budget of “leader dollars” which you can spend at your discretion.

Participants then saw three lists of traits, one at a time. The first two lists were prefaced by the following instructions:

Please design your ideal leader using the traits listed below. How many leader dollars would you spend for your ideal leader to possess each of these traits? For each trait, drag the bars to indicate how many leader dollars you would be willing to spend for your ideal leader to possess the trait in varying amounts. For example, if your ideal leader would be highly creative, you may want to spend $9-10 leader dollars on that trait. In contrast, if your ideal leader would be only a little extroverted, you may want to spend $0-1 leader dollars on that trait.

Participants then saw the list of traits, including a budget specification (e.g., “Your total budget is $60. You may not exceed this budget when designing your ideal leader.”) After rating all traits on a given list, participants were prompted to do this again with a different budget:

Now we would like you to try this again, only this time you have fewer leader dollars to spend on your ideal leader. For each trait, drag the bars to indicate how many leader dollars you would be willing to spend for your ideal leader to possess the trait in varying amounts.

These instructions were accompanied by a new budget specification (e.g., “Your total budget is $40. You may not exceed this budget when designing your ideal leader.”) The task instructions were the same for the two lists containing positive traits (e.g., competence/communality and assertiveness/communality). Finally, the instructions for the third list, which contained negative masculine and feminine stereotypes, read as follows:

Now we are interested in which characteristics you would not want your ideal leader to possess. How many leader dollars would you spend for your ideal leader not to possess each of these traits? For each trait, drag the bars to indicate how many leader dollars you would be willing to spend for your ideal leader not to possess the trait in varying amounts. For example, if you would strongly prefer that your ideal leader not be lazy, you may want to spend $9-10 leader dollars to avoid that trait. In contrast, if you have only a modest preference that your ideal leader not be forgetful, you may want to spend $0-1 leader dollars to avoid that trait.

These instructions were followed by budget specifications.

## Appendix B Study 2 Stimuli

Participants first read the following instructions, customized to condition. In the leader role condition, the text read:

Imagine you are leading a team on a special and important new project. As the leader, you are in charge of putting together a team of people to assist you in completing the project. You also determine what needs to be done in order to achieve your goals, and you assign tasks to your team members as you consider appropriate. As the leader, you also make sure team members follow your instructions and deliver in a timely manner, without missing any important deadlines. Ultimately, you are responsible for the final product, and it is your job to lead the team effort to realize your vision and complete the project successfully.

In the assistant role condition, participants read the following:

Imagine you are assisting a leader on a special and important new project. As an assistant, your job is to provide support to the team leader in completing the project. The team leader determines what needs to be done in order to achieve the team’s goals, and assigns tasks to you as appropriate. As an assistant, you follow the leader’s instructions, and you must deliver in a timely manner, without missing any important deadlines. Ultimately, the leader is responsible for the final product, and it is your job to help realize the leader’s vision and support and assist the leader to complete the project successfully.

After reading these role instructions, all participants read the following instructions prior to rating a series of traits:

Below is a list of traits and attributes. Please indicate how important each of them is to be successful in your role as (team leader / team assistant). In other words, consider how much each of these traits would help you fulfill your role as (team leader / team assistant).
